# SPARCL1 Enrichment at the Glioblastoma Invasive Front Is Consistent with Synaptogenic and Angiogenic Tumor Niches

**DOI:** 10.3390/ijms27094017

**Published:** 2026-04-30

**Authors:** JuliAnne E. Allgood, Torrance Johnson, Jessica E. Pullan

**Affiliations:** 1 School of Osteopathic Medicine, Rocky Vista University, Ivins, UT 84738, USA; 2 Department of Chemistry and Physics, Southern Utah University, Cedar City, UT 84720, USAjessicapullan@suu.edu (J.E.P.)

**Keywords:** SPARCL1, glioblastoma, infiltrating tumor

## Abstract

Astrocytes regulate key aspects of the neural microenvironment that can be co-opted by cancer to support tumor growth and invasion. Secreted protein acidic and rich in cysteine-like 1 (SPARCL1) is a matricellular glycoprotein expressed by astrocytes and stromal cells, whose expression varies across cancer types. While SPARCL1 is downregulated in many peripheral cancers, reports of its expression in gliomas, specifically glioblastoma (GBM), are inconsistent. The biological context underlying these divergent findings, and the role of SPARCL1 in GBM malignancy, remains unclear. Publicly available transcriptomic datasets from the Ivy Glioblastoma Atlas Project (Ivy GAP), GlioVis, and TCGA were analyzed to evaluate SPARCL1 expression across GBM cohorts. Spatially resolved gene expression data from Ivy GAP were used to assess SPARCL1 expression from defined tumor regions. Microarray and RNA sequencing datasets from GlioVis and TCGA, respectively, were used to assess SPARCL1 expression across whole-tumor samples. Spatial transcriptomics from Ivy GAP show SPARCL1 expression was upregulated along the leading edge and in infiltrating tumor regions. Microarray datasets showed greater SPARCL1 expression in tumors of astrocyte lineage as opposed to oligodendrocyte lineage. Bulk RNA sequencing showed high SPARCL1 expression in low-grade gliomas, which is consistent with astrocytic lineage, IDH mutation, and spatial averaging effects that might obscure regional associations. These findings demonstrate that SPARCL1 expression in GBM is shaped by tumor architecture, molecular classification, and microenvironment interactions. Enrichment of SPARCl1 at invasive tumor margins is consistent with prior studies linking SPARCL1 to neuron–glioma synapse formation and angiogenesis.

## 1. Introduction

Malignancies arising from glial cells, collectively termed gliomas, represent the most common primary malignant brain tumors in adults, with an incidence of approximately six per 100,000 individuals in the United States [1]. The most aggressive glioma subtype is glioblastoma (GBM), which is defined as a grade 4, IDH-wildtype, diffuse astrocytic glioma with distinct histologic or molecular criteria by the 2021 WHO classification guidelines [2]. GBM carries a poor prognosis, with a median survival of 12–15 months and a high chance of recurrence following surgical resection due to its highly aggressive, diffusely infiltrative nature [3,4,5].

Sequencing of GBM tumors and surrounding tissue has recently begun to elucidate the molecular determinants underlying GBM aggressiveness, cellular plasticity, and transcriptional state switching. These techniques allow for the identification of key genes that influence invasive behavior, treatment resistance, and disease progression. One emerging molecular candidate in this context is secreted protein acidic and rich in cysteine-like 1 (SPARCL1), a matricellular glycoprotein that has variable expression by astrocytes and stromal cells in development and disease states.

SPARCL1 is expressed throughout the body and contributes to cell adhesion, proliferation, and synaptogenesis during development [6,7]. In early neural development, SPARCL1 is highly expressed by radial glia and immature astrocytes, where it facilitates synaptic assembly and extracellular matrix (ECM) organization within the developing cortex [6]. SPARCL1 expression by radial glial cells helps terminate neuronal migration upon arrival at target cortical layers and promotes detachment from radial glial fibers through anti-adhesive properties, enabling proper cortical organization [8]. SPARCL1 also helps promote glutamatergic synapse formation and dendritic spine maturation through a yet unidentified signaling mechanism that does not involve neurexin and neuregulin [7,9]. Following development, SPARCL1 expression declines markedly, but expression can be increased after central nervous system (CNS) injury or in pathological states such as cancer, where it supports synaptic remodeling and network reorganization [6].

Outside of the CNS, SPARCL1 is highly expressed in quiescent endothelial cells, contributing to vascular stability and integrity [10]. High SPARCL1 expression is associated with blood vessel maturation and reduced endothelial proliferation and migration, whereas loss of SPARCL1 expression promotes endothelial activation, increased proliferation, and heightened vascular permeability [10,11]. SPARCL1 acts as a tumor suppressor outside of the CNS, and downregulation results in a greater chance of progression or metastasis [12,13,14,15].

In gliomas, SPARCL1 is significantly upregulated relative to non-tumor brain tissue and has been implicated in tumor infiltration by promoting angiogenesis and neuron–glioma synapses (NGS) formation in glioma cancer stem cell populations [16,17,18]. Notably, SPARCL1 expression has been reported to be highest in low-grade gliomas (LGGs) harboring isocitrate dehydrogenase (IDH) mutations [19]. Li et al. proposed a functional association between IDH mutation status and SPARCL1 upregulation, suggesting a potential mechanistic link between glioma metabolic reprogramming and SPARCL1-mediated microenvironmental remodeling; however, this relationship remains incompletely explored [19]. If such a link exists, lower SPARCL1 expression in GBMs would be expected due to its IDH-wildtype classification [20]. Contrarily, SPARCL1 is significantly upregulated in many GBM datasets compared with non-tumor tissue and has been shown in preclinical models to play a critical role in the generation and maintenance of cancer stem cell-derived glioma models [13,16,17,21].

The influence of SPARCL1 on GBM malignancy remains unresolved. This study aims to determine whether spatial transcriptomic, microarray, and bulk RNA sequencing datasets could account for the variable reports of SPARCL1 expression in gliomas and to evaluate whether tumor architecture, histologic subtype, or tumor grade are associated with differential expression. Using publicly available GBM datasets, SPARCL1 expression was novelly compared across spatial tumor regions (leading edge (LE), infiltrating tumor (IT), pseudopalisading cells around necrosis (PPC), and microvascular proliferation (MVP)), histologic tumor types (astrocytoma and oligodendroglioma), and tumor grades (low grade glioma (LGG), high grade glioma (HGG), and GBM) to determine the spatial and biological contexts in which SPARCL1 expression is enriched.

## 2. Results

### 2.1. Spatial SPARCL1 Expression Across GBM Tumor Regions

**Figure 1:**
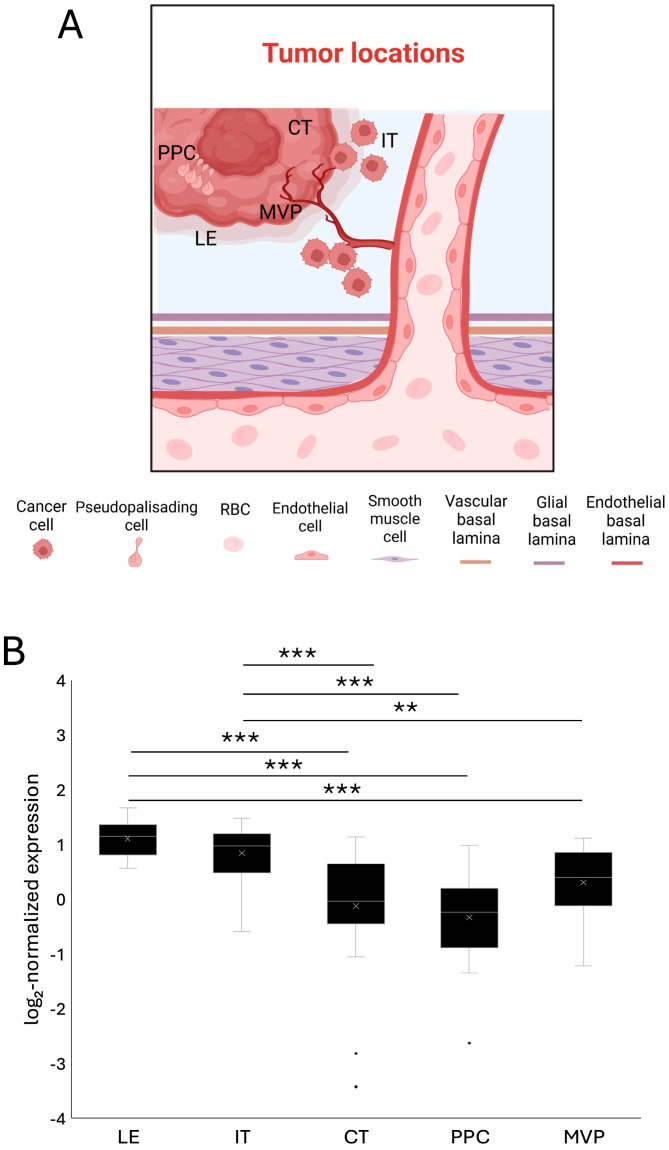
SPARCL1 expression is spatially determined in glioblastoma. (**A**) Schematic illustrating spatially defined tumor regions analyzed in the Ivy GAP dataset: LE, leading edge; MVP, microvascular proliferation; CT, cellular tumor core; IT, infiltrating tumor; PPC, pseudopalisading cells around necrosis. (**B**) Log2-normalized SPARCL1 expression across spatially annotated GBM regions in the Ivy GAP dataset. ** *p* < 0.01; *** *p* < 0.001. LE *n* = 19, IT *n* = 24, CT *n* = 30, PPC *n* = 24, MVP *n* = 25. Created in BioRender. JuliAnne Allgood (2026) https://app.biorender.com/illustrations/696d0bc834fa784263271e7a?slideId=47a069ea-7abb-4d9b-8e30-e0b9364e6652.

Figure 1A shows the spatial regions analyzed in Ivy GAP. Infiltrating tumor cells leave the main body of the tumor to enter the surrounding parenchyma, causing transformation of healthy glia, recruitment of vasculature, and new tumor growth if not removed. The leading edge of the tumor also displays some of these characteristics and will be non-contrast enhancing in a majority of MRIs because it is not yet a solid tumor mass. Regions of microvascular proliferation are areas where new blood vessels are being formed and directed to oxygen-starved tumor cells. The cellular tumor core is the main cell-dense region of the glioblastoma tumor, where necrosis often occurs. Pseudopalisading regions around the cellular tumor core are areas of cells attempting to move away from the core to more oxygen- or nutrient-rich areas.

Spatial transcriptomic analysis of these regions demonstrated significant regional variation in SPARCL1 expression. Expression levels were significantly greater in LE (log2 normalized expression = 1.10) and IT (log2 normalized expression = 0.83) regions compared to CT (log2 normalized expression= −0.13; *p* < 0.001; *p* < 0.001), PPC (log2 normalized expression= −0.33; *p* < 0.001; *p* < 0.001), and MVP (log2 normalized expression = 0.30; *p* < 0.001; *p* = 0.006) regions (Figure 1B). There were no statistical differences between CT, PPC, and MVP regions, indicating that SPARCL1 is not uniformly distributed across GBM tumors and instead varies by tumor microenvironment and cellular function.

### 2.2. SPARCL1 Expression Across Histologic Glioma Subtypes

**Figure 2:**
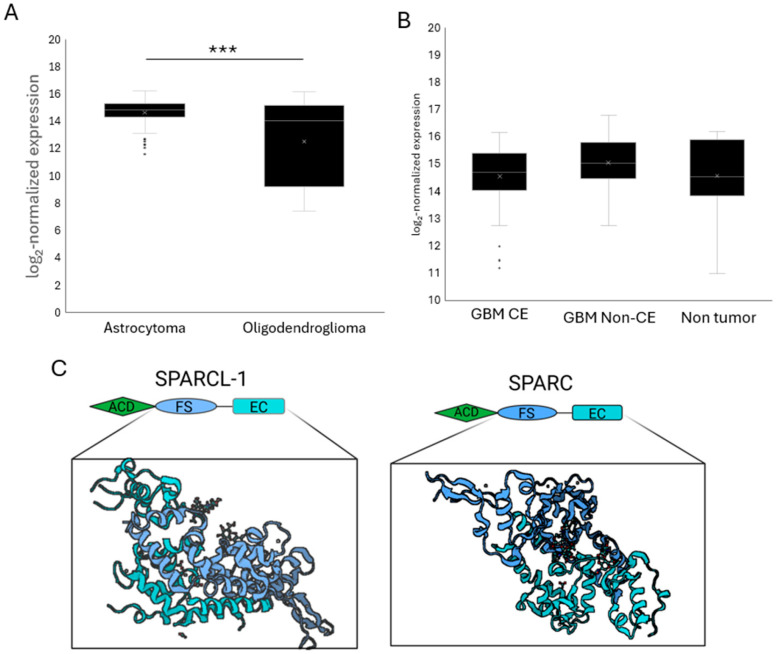
SPARCL1 expression varies with histologic composition in glioblastoma. (**A**) Microarray-based SPARCL1 expression in samples classified as GBM and further stratified by histologic subtype. *** *p* < 0.001. Astrocytoma *n* = 154, oligodendroglioma *n* = 62. (**B**) Microarray-based SPARCL1 expression in GBM samples categorized as contrast-enhancing (CE), non–contrast-enhancing (non-CE), or non-tumor tissue. GBM CE *n* = 38, GBM nonce *n* = 37, nontumor *n* = 32. (**C**) Schematic illustrating the substantial sequence homology between SPARCL1 and SPARC (~60%).

Data from histologically defined GBM subtypes show that SPARCL1 expression was significantly higher in astrocytoma compared with oligodendroglioma. Astrocytomas demonstrated the highest overall expression levels (log2 normalized expression = 14.66), which were significantly greater than oligodendroglioma (log2 normalized expression = 12.54) (*p* < 0.001) (Figure 2A). This finding is consistent with SPARCL1 release from astrocytes more than from other glia of the CNS.

When comparing microarray data from CE (log2 normalized expression = 14.56) and non-CE (log2 normalized expression = 15.05) GBM samples, a trend toward increased SPARCL1 expression was observed in the non-CE group; however, this difference did not reach statistical significance (*p* = 0.187). Although not statistically significant, this trend aligns with the spatial enrichment of SPARCL1 observed at infiltrating tumor margins (Figure 2B).

Interpretation of microarray-based expression data must also consider probe design. SPARCL1 shares significant homology (~60%) with the related matricellular protein SPARC (Figure 2C). Although these proteins differ structurally, their domain similarities can complicate probe specificity, potentially introducing variability in microarray measurements.

### 2.3. SPARCL1 Expression Across Tumor Grades Using RNA Sequencing Data

**Figure 3:**
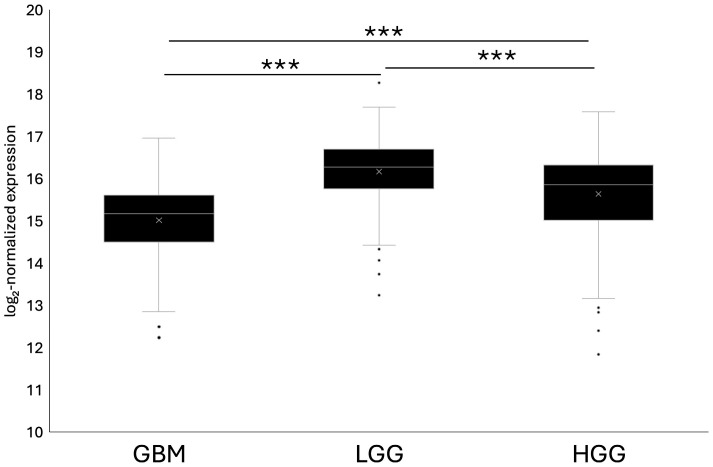
SPARCL1 expression assessed by RNA sequencing across tumor grades. Log2-normalized SPARCL1 expression derived from TCGA RNA sequencing datasets, compared across GBM, LGG, and HGG. *** *p* < 0.001. GBM *n* = 156, LGG *n* = 226, HGG *n* = 244.

SPARCL1 expression across tumor grades varied significantly, with GBM samples showing the lowest expression and LGG showing the highest expression, with a 1.07 fold change between them (Figure 3). SPARCL1 expression in LGG was significantly greater than both HGG and GBM, and HGG was significantly greater than GBM as well. These findings highlight differences in SPARCL1 expression across glioma grades when assessed using bulk RNA sequencing datasets but ignore the nuance of spatially defined region variation and tumor cell lineage.

## 3. Discussion

SPARCL1 expression in GBM appears dependent on spatial boundaries, tumor cell lineage, and tumor grade. Spatial transcriptomic analysis demonstrated enrichment of SPARCL1 transcripts in the LE and IT regions of GBM, with comparatively lower expression in PPC, CT, and MVP regions. This spatial heterogeneity helps reconcile previously conflicting reports of both increased and decreased SPARCL1 expression in GBM and underscores the importance of spatial tumor context when interpreting transcriptomic datasets [6,13,17,21]. Consistent with recent studies linking SPARCL1 to neuron–glioma synapse (NGS) formation [19], our findings demonstrate enrichment at invasive tumor margins with statistically significant differences across defined regions. These spatial patterns likely reflect not only tumor architecture but also underlying transcriptional programs, as prior work has shown SPARCL1 enrichment in invasive, stem-like glioma populations [6]. By examining SPARCL1 across anatomically defined tumor compartments and integrating multiple transcriptomic datasets, this study provides a framework for understanding how its expression varies across GBM.

**Figure 4:**
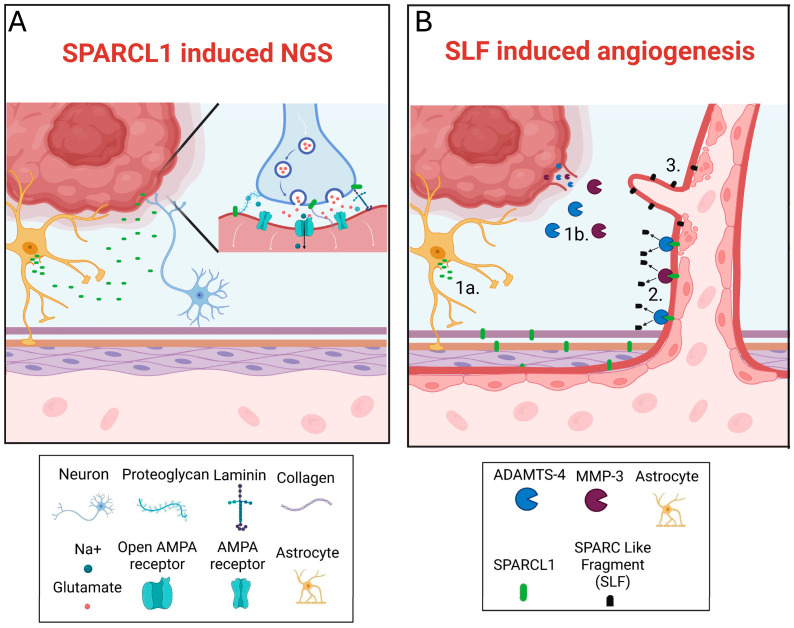
Proposed model of SPARCL1 function in GBM. (**A**) SPARCL1 contributes to neuron–glioma synapse (NGS) formation through stabilization of the perisynaptic ECM, including interactions with collagen, proteoglycans, and laminin. (**B**) SPARCL1 contributes to angiogenesis. SPARCL1 is released from astrocytes (1a), while ADAMTS-4 and MMP-3 proteases are expressed within the GBM tumor microenvironment (1b). Proteolytic cleavage of SPARCL1 within the ECM (2) generates SLFs, which disrupt ECM integrity (3) and facilitate endothelial sprouting toward GBM tumors.

Localization of high levels of SPARCL1 transcripts in LE and IT regions, as well as in non-CE enhancing regions, aligns with its established roles in ECM organization and synaptogenesis within the CNS (Figure 4A) [13,22]. During development and injury response, SPARCL1 is expressed by astrocytes and radial glia, where it facilitates synaptic assembly through matricellular ECM remodeling rather than direct receptor interactions [7,8]. Enrichment of SPARCL1 in infiltrative GBM compartments, therefore, supports a model in which tumor cells exploit developmental ECM programs to integrate into surrounding neural tissue (Figure 4A). These regions correspond to sites of NGS formation, suggesting that SPARCL1 may contribute to the permissive perisynaptic microenvironment supporting neuron–tumor coupling rather than functioning as a direct synaptic ligand. This observation is particularly relevant given the diffuse infiltration of GBM and the contribution of NGS-mediated glutamatergic signaling to tumor proliferation and survival through MAPK and mTOR pathway activation [23,24,25]. By organizing ECM and synaptogenic niches at invasive tumor margins, SPARCL1 expression may facilitate tumor–microenvironment interactions, promoting both local invasion and persistence following surgical resection.

In contrast, relatively low SPARCL1 transcript levels in MVP regions suggest that angiogenic effects attributed to SPARCL1 may depend more on post-translational processing than transcriptional abundance. Proteolytic cleavage by ADAMTS-4 and MMP-3 generates SLFs that have been associated with ECM remodeling, endothelial migration, and vascular sprouting in glioma models (Figure 4B) [16,18,22]. This distinction may explain why SPARCL1 transcript enrichment is observed primarily in infiltrative regions, whereas angiogenic activity becomes more prominent in higher-grade diseases characterized by increased protease activity.

Histologic classification of tumor cell lineage further supports a context-dependent role for SPARCL1. Higher expression in astrocyte-derived tumors relative to oligodendrocyte-derived tumors is consistent with developmental patterns of SPARCL1 expression in the CNS. Interpretation of microarray-based expression data, however, must consider important technical limitations. Unlike spatial transcriptomic approaches, microarray analyses rely on probe binding to RNA extracted from whole tumor homogenates, which can obscure spatial relationships within heterogeneous tumors. This limitation is particularly relevant for SPARCL1, which shares substantial sequence homology with the related matricellular protein SPARC. Similar domain structures may complicate probe specificity and contribute to variability in microarray-based detection, further highlighting the importance of spatial context when interpreting SPARCL1 expression in GBM.

RNA-sequencing analysis from the TCGA dataset demonstrates higher SPARCL1 expression in LGG compared with GBM. Several factors are likely to contribute to this difference. Prior literature suggests SPARCL1 transcription is greatest during earlier stages of tumor progression and in IDH-mutant tumors, both of which are associated with greater neural integration and differentiation capacity [26]. Consistent with this, LGGs typically exhibit diffuse infiltration with relatively preserved neural architecture, a context in which SPARCL1-mediated ECM organization and synaptogenic niche formation may be advantageous. Thus, elevated SPARCL1 expression in LGG likely reflects astrocytic lineage programs and IDH-associated transcriptional states rather than invasion alone, whereas in GBM, SPARCL1 expression appears to be spatially restricted to infiltrative tumor regions where it may support invasion and tumor–microenvironment interactions.

Methodological factors may also influence this apparent expression difference. Because RNA sequencing datasets are derived from whole-tumor homogenates, spatially restricted gene expression patterns are averaged across heterogeneous tumor regions. Genes such as SPARCL1, which show spatial enrichment within IT and LE compartments, may therefore appear comparatively reduced when assessed using bulk RNA sequencing approaches.

Differences in diagnostic classification may also contribute to the observed variation in SPARCL1 expression between LGG and GBM cohorts. The 2021 WHO framework no longer categorizes all HGGs as GBMs and instead incorporates molecular features such as IDH mutation status [20]. Earlier datasets, including portions of TCGA, were generated under less restrictive diagnostic criteria, emphasizing histologic grade over molecular classification [27]. Consequently, tumors previously labeled as GBM may encompass a broader range of molecular and histologic compositions than those classified under current criteria.

These considerations highlight several limitations inherent to public transcriptomic datasets, including reliance on bulk RNA sequencing, evolving classification frameworks, and variable sample sizes. Future studies integrating spatial transcriptomics, proteomics, and functional validation will be important for clarifying the role of SPARCL1 within the glioma TME. Nonetheless, the present findings emphasize the importance of spatial and developmental context when interpreting SPARCL1 expression and provide a framework for reconciling its seemingly divergent roles in glioma biology.

## 4. Materials and Methods

### 4.1. Data Sources and Cohort Selection

Publicly available transcriptomic datasets were queried to evaluate SPARCL1 expression in GBM. Gene expression data were obtained from GlioVis [28], an interactive visualization platform that aggregates bulk RNA sequencing and microarray datasets from glioma cohorts, including The Cancer Genome Atlas (TCGA), the Chinese Glioma Genome Atlas (CGGA), Gene Expression Omnibus (GEO), and additional published studies. To avoid cross-platform bias, datasets were separated by expression modality (microarray versus RNA sequencing), and comparisons were performed only within the same platform. All expression values were log_2_-normalized prior to analysis across all platforms and datasets.

Microarray datasets represented the largest portion of cohorts available in the GlioVis platform, and all datasets containing SPARCL1 expression data were initially examined (GEO, GBM, MD Anderson, Tumor Bank, French Glioma, and Clinical Institution cohorts). These datasets represent multiple independent GBM cohorts generated at different institutions and time points. Each dataset was therefore evaluated for batch effects based on sample size and expression distribution before selecting a dataset for downstream analyses (Supplementary Figure S1). Significant differences in SPARCL1 expression were observed across datasets (Supplementary Figure S1), consistent with known sources of variability including probe design, tissue acquisition site, sample age, and evolving WHO classification criteria. The datasets with the GSM identifier derived from GEO were selected due to their large sample size (*n* = 599). SPARCL1 microarray expression was subsequently compared with samples labeled non-tumor brain tissue, contrast-enhancing (CE) tumors, and non-enhancing tumors (non-CE), as well as across histologically defined cell types (astrocytoma and oligodendroglioma).

Spatially resolved transcriptomic data were obtained from Ivy GAP [16], which provides region-specific gene expression profiles from human GBM specimens. Only one dataset had been analyzed for SPARCL1 expression in Ivy GAP. Spatial SPARCL1 expression patterns were evaluated using Ivy GAP data by comparing expression across defined tumor regions, including the LE, IT, CT, PPC, and MVP. RNA sequencing-based SPARCL1 expression was analyzed using TCGA data and compared across GBM, low-grade glioma (LGG), and high-grade glioma (HGG) groups.

### 4.2. Statistics

Statistical comparisons between groups were performed using non-parametric tests due to non-normal expression distributions. Differences in SPARCL1 expression were assessed using a Kruskal–Wallis test, followed by post hoc pairwise comparisons where appropriate. Statistical significance was defined as *p* < 0.05. All statistical analyses were performed using SPSS version 31. Where applicable, sample sizes have been included in figure legends.

## 5. Conclusions

In summary, this study demonstrates that SPARCL1 expression in GBM is highly context dependent, with pronounced enrichment at the LE and IT regions rather than uniform upregulation across the tumor mass. These spatial patterns reconcile prior conflicting reports and support a model in which SPARCL1 contributes to glioma progression through region-specific ECM organization, facilitation of NGS formation, and post-translational regulation of angiogenesis. Higher SPARCL1 transcript levels observed in LGG likely reflect astrocytic lineage programs, IDH mutation-associated transcriptional states, and reduced spatial averaging. In GBM, localized SPARCL1 ex-pression coupled with increased proteolytic processing may drive invasive and angiogenic behavior. Overall, these results provide a unifying framework for understanding the seemingly paradoxical roles of SPARCL1 across glioma grade and tumor compartments and provide a model by which these results can be explained.

## Data Availability

All data are publicly available through Ivy Gap (https://glioblastoma.alleninstitute.org (accessed on 12 December 2025)) and GlioVis (https://gliovis.bioinfo.cnio.es (accessed on 12 December 2025)).

## Supplementary Materials

The following supporting information can be downloaded at: https://www.mdpi.com/article/10.3390/ijms27094017/s1.

## Abbreviations

The following abbreviations are used in this manuscript:

CNS	Central nervous system
SPARCL1	Secreted protein acidic and rich in cysteine-like 1
GBM	Glioblastoma
ECM	Extracellular matrix
NGS	Neuron-glioma synapse
IDH	Isocitrate dehydrogenase
LGG	Low-grade glioma
HGG	High-grade glioma
LE	Leading edge
IT	Infiltrating tumor
PPC	Pseudopalisading cells around necrosis
MVP	Microvascular proliferation
CT	Cellular tumor core
