# Supplementary figures and images for: SPARCL1 Enrichment at the Glioblastoma Invasive Front Is Consistent with Synaptogenic and Angiogenic Tumor Niches
Source: Int J Mol Sci. 2026 Apr 30;27(9):4017. doi: 10.3390/ijms27094017 (PMC13164218; doi:10.3390/ijms27094017)

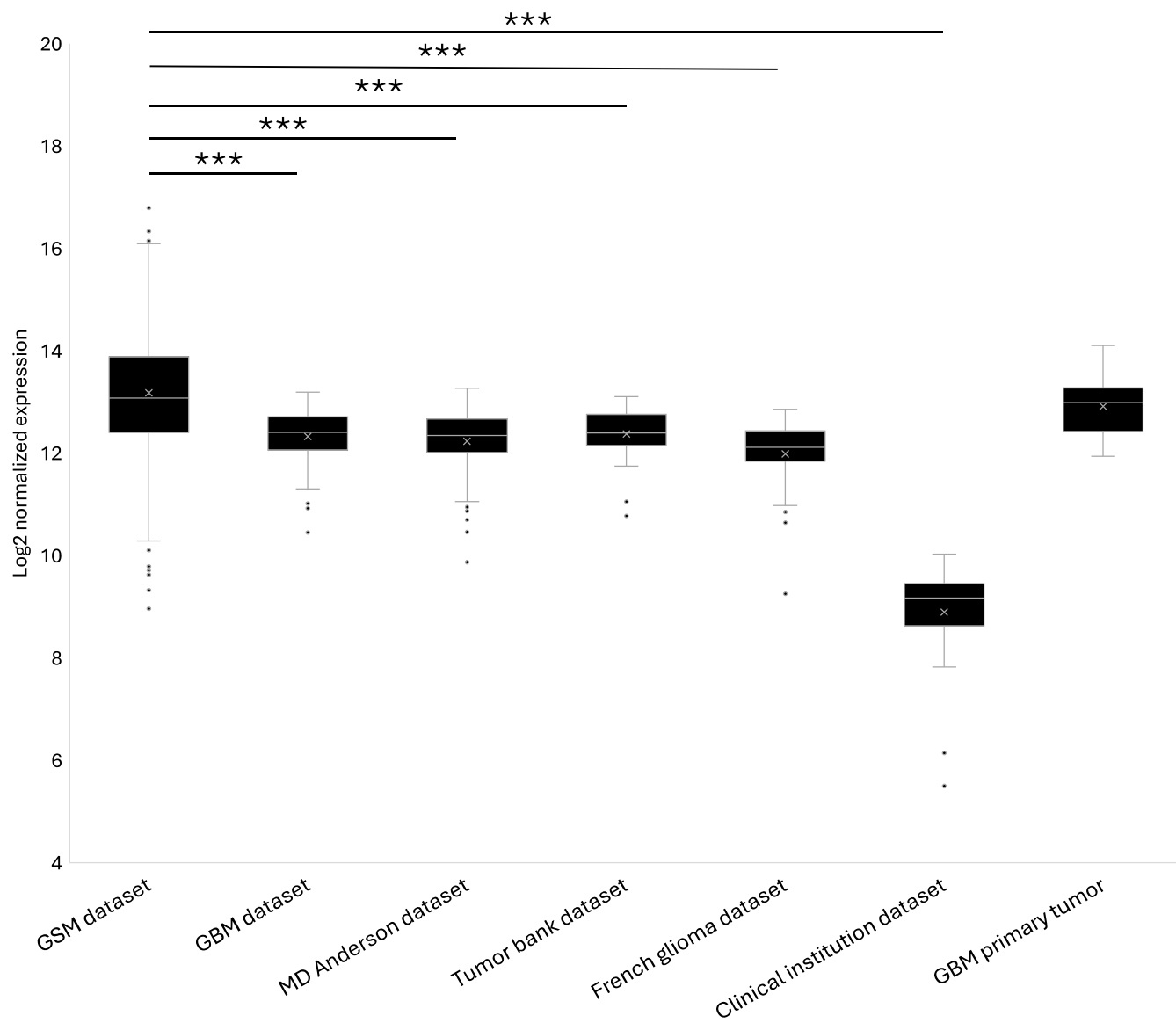

Supplement: Supplementary file 1 [file ijms-27-04017-s001.zip › ijms-4237364-Figure S1.pdf]
